# Quality Indexes of the ECG Signal Transmitted Using Optical Wireless Link

**DOI:** 10.3390/s23094522

**Published:** 2023-05-06

**Authors:** Amel Chehbani, Stephanie Sahuguede, Anne Julien-Vergonjanne, Olivier Bernard

**Affiliations:** 1 XLIM Laboratory, UMR CNRS 7252, University of Limoges, 87000 Limoges, France; amel.chehbani@unilim.fr (A.C.); anne.julien-vergonjanne@unilim.fr (A.J.-V.); 2 MOVE Laboratory, University of Poitiers, 86000 Poitiers, France; olivier.bernard@univ-poitiers.fr

**Keywords:** electrocardiogram (ECG) monitoring, outage probability, optical wireless communications (OWC), signal quality indexes (SQIs)

## Abstract

This work relates to the quality of the electrocardiogram (ECG) signal of an elderly person, transmitted using optical wireless links. The studied system uses infrared signals between an optical transmitter located on the person’s wrist and optical receivers placed on the ceiling. As the elderly person moves inside a room, the optical channel is time-varying, affecting the received ECG signal. To assess the ECG quality, we use specific signal quality indexes (SQIs), allowing the evaluation of the spectral and statistical characteristics of the signal. Our main contribution is studying how the SQIs behave according to the optical transmission performance and the studied context in order to determine the conditions required to obtain excellent quality indexes. The approach is based on the simulation of the whole chain, from the raw ECG to the extraction process after transmission until the evaluation of SQIs. This technique was developed considering optical channel modeling, including the mobility of the elderly. The obtained results show the potential of optical wireless communication technologies for reliable ECG monitoring in such a context. It has been observed that excellent ECG quality can be obtained with a minimum SNR of 11 dB for on–off keying modulation.

## 1. Introduction

With age, cardiovascular diseases are one of the most common reasons for hospitalization. However, since the symptoms of cardiac pathologies can appear suddenly and sporadically in the elderly, too late hospitalization can lead to an unfavorable vital prognosis. It is therefore important to have the means to detect the early signs of heart diseases (like heart failure, heart stroke, arrhythmias, etc.).

One solution is to implement continuous, long-term monitoring of the electrical heart activity, represented by an electrocardiogram (ECG) signal. For this purpose, we can use wearable devices such as widely used wired Holter monitors, which provide a high-quality ECG signal. However, they are generally limited to 24–48 h of continuous use without real-time data transmission. In addition, wires can cause discomfort for people during their daily activities [1]. In contrast, implantable devices provide continuous real-time monitoring but require expensive surgical procedures and pose risks of infection [2]. There is therefore a need for wireless solutions offering portability, mobility, and flexibility.

Advances in hardware, software, and wireless communication technologies have led to the development of new, convenient, and affordable devices [3] that allow ECG signal monitoring. Today, these devices are wearable systems [4], generally using RF communication technologies. However, with the proliferation of connected objects in people’s daily environments, the risks of RF interference and security vulnerabilities can limit the quality of ECG diagnoses.

Today, optical wireless communications (OWC) technology offers connectivity in environments where RF deployment and/or performance are limited [5,6,7,8]. Indeed, this technology provides an RF-free and therefore interference-free environment, where data can be transmitted using infrared (IR), visible light, or the ultraviolet band without licensing requirements. It has high efficiency with respect to the confidentiality of transmissions thanks to the confinement of the light beams. In addition, it is a sustainable and inexpensive alternative to RF because it is possible to exploit existing lighting in visible light communication (VLC) as well as photovoltaic components to harvest energy and simultaneously decode optical signals [9]. As a result, OWC technology is now recognized as a solution for Internet of Things (IoT) applications [10], especially for medical data monitoring [11,12,13,14,15,16], ECG signal transmission [17,18,19,20,21,22,23,24,25,26,27,28,29,30,31], and the connectivity of implantable medical devices [32].

Nevertheless, whatever the communication technology used, the quality of the ECG signal is the main issue for good interpretation and, therefore, a good diagnosis. In addition to acquisition noise and transmission channel impairments, various disturbances such as motion artifacts, power line interference, and signal electromyography (EMG) can affect signal quality [33]. Thus, the evaluation of ECG devices has received considerable attention from researchers and has become a hot topic. The quality of an ECG may be studied at different levels, from signal acquisition to reconstruction, but also at the level of transmission via a wireless link. Several studies have focused on the analysis of communication performance using conventional physical layer metrics such as signal-to-noise ratio (SNR) and bit error rate (BER), while others have analyzed signal quality indexes (SQIs) based on the extraction of the received ECG signal characteristics. In 2008, Li et al. [34] were among the first to develop signal quality metrics based on ECG feature extraction. Since the success of the PhysioNet Cardiology challenge in 2011 [35], various SQIs have been developed and improved, exploiting the morphological, spectral, temporal, and statistical characteristics of the signal; these have been widely used in combination with automatic algorithms and artificial intelligence tools, the aim being to generate alerts for events and situations requiring immediate medical attention [36,37,38,39,40,41,42,43,44]. In this work, we use SQIs for the evaluation of ECG signal quality relative to the optical transmission conditions. We are particularly interested in spectral and statistical SQIs. Spectral ones allow a detailed analysis of the overall frequency spectrum of the ECG signal to detect and quantify various types of artifacts. Statistical SQIs characterize the distribution of an ECG signal, which provides information about the presence of outliers and extremes in the signal, indicating anomalies, artifacts, or other sources of interference.

To the best of our knowledge, the impact of the wireless communication channel on the received ECG SQIs has not been studied before, either in RF or in OWC. In a previously published work [31], we presented the first results of our research focusing on spectral SQIs and considering optical wireless transmission of the ECG. In this article, we extend and improve this first study by proposing an approach whereby it is possible to determine the OWC link conditions required to obtain acceptable spectral and statistical SQIs. The rest of the paper is organized as follows. In the following section, we discuss related work and our motivation for this research. Section 3 describes the environment and OWC channel modeling. In Section 4, we detail the ECG transmission chain. Then, Section 5 presents the used SQIs for the ECG signal and the proposed methodology. Finally, we analyze the impact of the optical channel on the ECG SQIs and present our results. Finally, Section 6 is the conclusion.

## 2. Related Work

Although OWC technology can be suitable for safe, remote ECG signal monitoring, few works have been published on this topic [17,18,19,20,21,22,23,24,25,26,27,28,29,30] (see Table 1). The first observation from Table 1 is that the majority of work in this field has been carried out in the visible band (VLC technology), essentially by conducting experimental approaches [17,18,19,20,21,22,23,24,25,27,29]. These studies demonstrated the potential of OWC in the visible range for remote transmission of ECG. However, using visible light for a user-worn transmitter can be uncomfortable. IR transmission, which is more suitable in terms of comfort, has nevertheless been the subject of fewer studies [26,28,30]. For example, in [30], we investigated IR technology for baby ECG monitoring because, in this case, visible light can be very disturbing.

Another remark concerns the studied link configurations, which are, for the first published papers, static, point-to-point, line-of-sight (LOS) links [17,18,19,20,21,22,23,26,29]. As LOS signals have a high blockage probability when the link is moving, this approach is not suitable for ECG monitoring of mobile patients. To address this problem, other approaches have shown the benefit of using non-LOS links, which are conventionally used in indoor OWC mobile systems [24,25,28,30]. Another solution is to deploy hybrid optical/radio links, as in [27], with optical camera communication (OCC); however, this comes at the cost of a more complex system, requiring an effective handover mechanism to guarantee performance.

As shown in Table 1, the efficiency of monitoring systems is evaluated through low layer metrics such as emitted power, receiver sensitivity, SNR, BER, outage probability, or PER. In the upper layer, the quality of the recovered ECG signal is assessed in almost all cases by comparing the shapes of the transmitted and received ECG and checking the heart rate (HR) metric. In addition, in some studies, the signal quality was not evaluated [21,27]. However, it is well known that SQIs are common metrics used to evaluate ECG, which can be calculated from different signal features, i.e., temporal, spectral, morphological, and statistical. This is an approach to determine if the signal is useful for further processing and to identify trustworthy events, thereby avoiding misinterpretations.

Thus, to discuss the reliability of ECG in detecting cardiac symptoms during regular follow-up, it is essential to assess the impact of optical transmission reliability on SQIs. Among the published works, the impact of OWC on the ECG SQIs, in particular, the spectral and statistical characteristics of interest, has not been analyzed, except in [29], where the quality of a VLC-based ECG signal was evaluated in terms of RR peak accuracy as a function of receiver sensitivity.

We have previously adopted the SQI feature extraction method [30] for the case of ECG monitoring of a baby. The results showed that a minimum SNR of 10 dB is necessary to achieve sufficient ECG quality. Here, we consider another context for a much more time-varying channel. We consider an elderly person equipped with an ECG sensor worn on the wrist, moving in an indoor environment. To collect ECG data, a receiver system is placed on the ceiling of the environment. In this context, the quality of the received signal varies over time due to the variation of the transmission channel as the person moves around. Therefore, our goal is to study the requirements for optical wireless transmission when monitoring an elderly person, in order to satisfy the SQI values required for reliable ECG signal analysis. This is an extension of recently published work [31]. In the study presented here, we consider the impact of the OWC channel on the spectral SQIs but also on the statistical ones. Previously, we considered the SQI identification benchmarks defined in the literature; however, these are not properly adapted to the studied context and to the evaluation method to ensure an accurate analysis. Therefore, we follow an improved methodology by computing the SQIs before and after transmission and by considering the impact of the temporal windowing used. Additionally, we discuss the robustness of SQIs with respect to the emitted optical power, which is important when considering a portable system. Last but not least, this work offers a general method for assessing the viability of an optical ECG wireless monitoring system in indoor environments by looking at how well it performs in terms of ECG transmission and quality reliability, whatever the setting and the assessment indexes employed.

## 3. System Overview

### 3.1. Context

**Figure 1:**
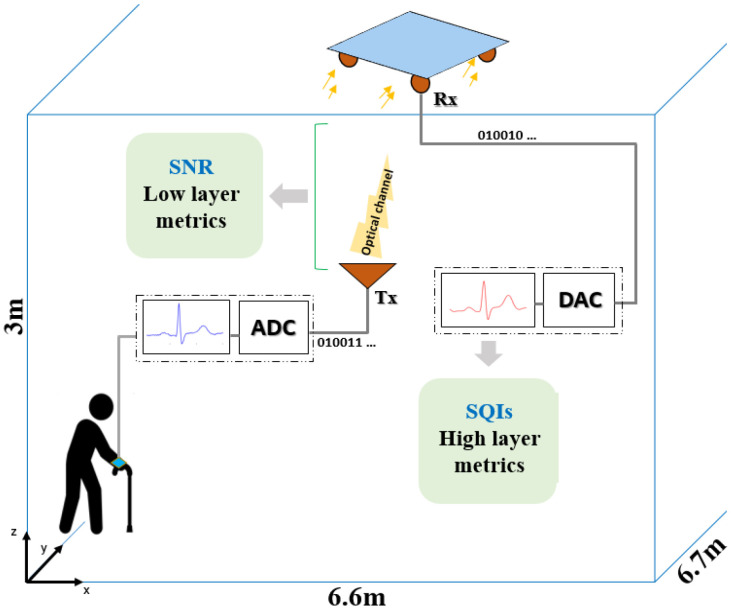
Context illustration.

The system to monitor the ECG signal of an elderly individual walking in a room is illustrated in Figure 1, based on the work presented in [13]. The environment is an empty room with dimensions of $6.6\times 6.7\times 3\;m^{3}$. All room surfaces are considered Lambertian, with reflectivity ρ=0.8.

The elderly person was equipped with an ECG device linked to an optical wireless emitter (Tx) worn on the wrist. The ECG to be transmitted over the proposed optical channel is defined in Section 4. Four optical receivers were placed at the ceiling, collecting data during the person’s random walk. The receivers (Rx), each including a photodiode with a physical surface of 34.5 mm² and a field of view (FOV) of 45°, were fixed at the corners of a panel placed in the center of the room ceiling. Each receiver was oriented 45° from the ceiling.

Moreover, we used the switching combining method, which consists of processing data with a higher signal strength. It was verified in [13] that this configuration provides good communication coverage. Digital-to-analog conversion (DAC) was then applied to restitute the received ECG signal, the quality of which was evaluated by calculating high-layer metrics, i.e., SQIs.

### 3.2. Channel Modeling

A line-of-sight (LOS) link is not always possible due to walking. Thus, the contributions of optical reflections on the different surfaces providing the non-LOS (NLOS) link must be taken into account to determine *h*(*t*)*,* the impulse response of the channel. This was done by considering three reflections per optical beam through Monte Carlo ray-tracing (MCRT) simulations using dedicated software called RapSor, developed in our laboratory [45].

**Figure 2:**
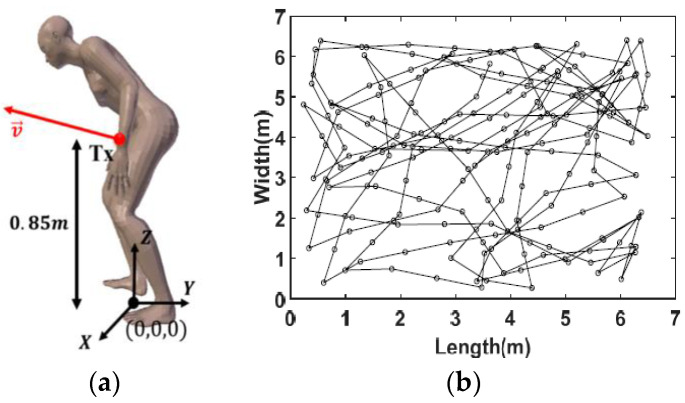
(**a**) Figure of elderly person, (**b**) example of the person’s travel trajectory using the RW mobility model [13].

With this aim, the elderly person was modeled using the 3D body model shown in Figure 2a, considering the torso leaning forward and the body surface reflectivity coefficient (ρ=0.1). The optical transmitter Tx is illustrated in Figure 2a. It was placed on the wrist of the elderly person at X=0.22 m, Y=0.14 m, Z=0.88 m. Thus, the optical source of the Tx had an orientation that varied according to the movements of the arm during walking (refer to Figure 2a).

As explained in [13], the specific movements of the elderly person during walking were discretized from walking cycle recordings considering a speed of 0.5 m/s and a step of 20 cm in length, that is, a step duration $T_{step}=0.4\;s$. The movement of the whole body was described using a random walk mobility model, illustrated as an example in Figure 2b.

Through the MCRT simulations, the impulse responses corresponding to each link resulting from movements and mobility were obtained, from which the static gains $H_{0}$ could be calculated as:

(1) $$H\left(0\right)=\int_{-\infty }^{+\infty }h\left(t\right)dt=H_{0}$$

**Figure 3:**
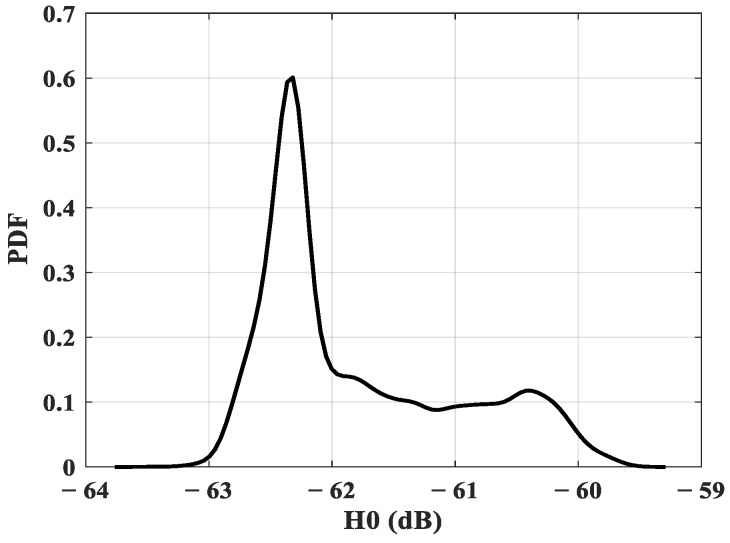
Static gain $H_{0}$ distribution.

Figure 3 shows the distribution of the gains $H_{0}$ considering an optical source with a half-power angle of 45° and assuming that the elderly person occupied 10,000 positions over approximately 1 h. The distribution shows a probable maximum gain value of −60.6 dB.

Then, considering the conditions of the studied configuration, we observed that the static channel gain varied between a minimum value of −62.98 dB and a maximum value of −55.12 dB. This range represents the attenuation undergone by the signal during LOS and NLOS optical transmissions.

To illustrate its impact on the ECG signal during walking, we have developed a simulation chain, which is presented in the following section.

## 4. Optical Wireless ECG Transmission

### 4.1. Transmission Chain

**Figure 4:**
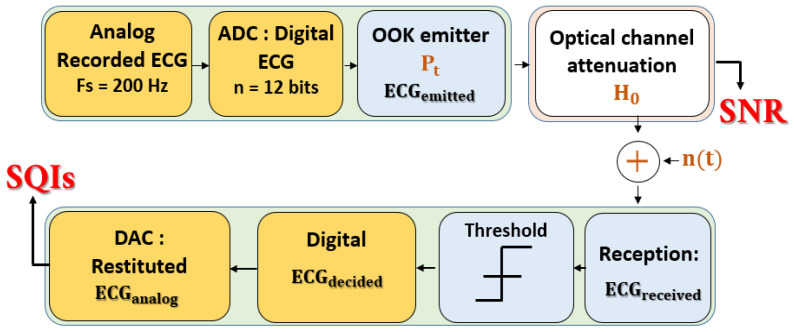
Simulation transmission chain.

The chain represented in Figure 4 was used to simulate the transmission of a raw ECG signal using OWC technology. The raw signal, used as described in [46], was recorded from a healthy adult subject using a Powerlab system with a sampling frequency $F_{s}$ of 200 Hz and was affected by a 60 Hz power-line frequency noise. A notch filter was used to remove this artifact.

The block analog-to-digital (ADC) process shown in Figure 4 consists of digitalizing the ECG signal with n=12 bits, which is a recommended resolution for ECG signals. Therefore, a minimum data rate $R_{min}$ of $F_{s}\times n=2.4\;kbits/s$ is required for real-time transmission and analysis. This conforms to the specifications guaranteeing the reliability of continuous wireless ECG monitoring [47]. Moreover, considering this data rate, we verified that channel delay dispersion and, therefore, inter-symbol interference, are negligible [13].

Then, the transmitter block allowed us to shape the signal according to the classic on–off keying (OOK) modulation by considering an average optical emitted power $P_{t}$. This modulation consisted of emitting an optical pulse if the bit to be transmitted was “1” and no pulse if the bit was “0”. Next, the emitted ECG signal underwent optical channel attenuation, characterized by the static DC gain $H_{0}$, as determined by MCRT simulations.

We also took into account the additive white Gaussian noise n(t), mainly due to shot noise related to the induced ambient current $I_{B}$, characterized by its variance $\sigma^{2}$, which may be expressed by:

(2) $$\sigma^{2}=2qI_{B}B$$

where q is the electron quantum charge and B is the modulated signal bandwidth equal to the data rate $R_{b}$ for the OOK modulation.

$I_{B}$ is the photocurrent characterizing the ambient optical noise at the optoelectronic sources set to 200 µA in this study [31].

Upon reception, the noisy and attenuated received ECG signal is directed to a decision system. For OOK modulation, considering average emitted power $P_{t}$ and equiprobable transmission, we used a decision system with a threshold defined as:

(3) $${Th}_{OOK}=P_{t}\times H_{0}$$

According to ${Th}_{OOK}$, the decided bits of the ECG signal (${ECG}_{decided}$) were obtained as follows:

(4) $${bit}_{decided}=\left\{\begin{array}{c} 1\;\;if\;{bit}_{received}\geq {Th}_{OOK}\\ 0\;\;if\;{bit}_{received}<{Th}_{OOK} \end{array}\right.$$

Finally, the restituted analog signal ${ECG}_{analog}$ was obtained using the last block (DAC) of the chain in Figure 4. The SQIs may be calculated from this signal.

As previously mentioned, optical transmission channels are commonly evaluated using low-layer metrics such as the SNR [5], defined for OOK as:

(5) $${SNR}_{OOK}=\frac{P_{t}^{2}R^{2}H_{0}^{2}}{qI_{B}R_{b}}$$

whereR is the responsivity of the photodiode set to 1A/W in this work. 

The BER performance is then obtained using:

(6) $$BER=0.5\times erfc(\sqrt{SNR/2})$$

### 4.2. Impact of Channel Behaviour on ECG during Time

**Figure 5:**
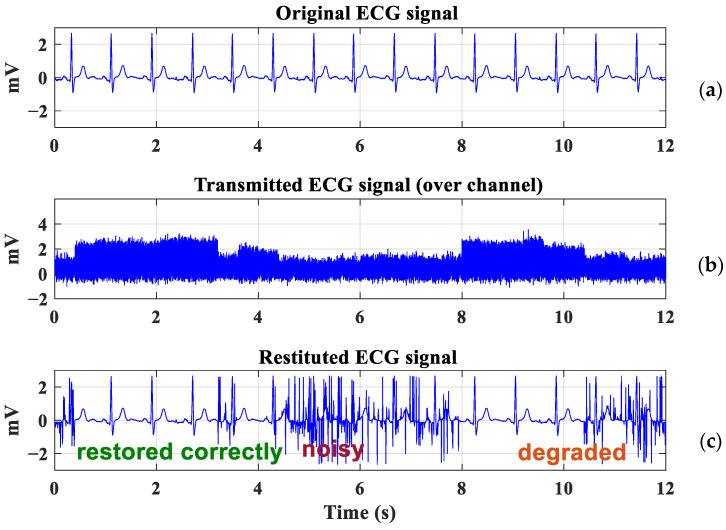
ECG transmission: (**a**) Original ECG signal, (**b**) Attenuated ECG signal, (**c**) Restituted ECG signal.

In order to illustrate the impact of channel behavior on the ECG signal, thanks to the chain detailed in Section 4.1, we have plotted in Figure 5 an example of ECG transmission during 12 s of monitoring, corresponding to 30 positions of the elderly subject, i.e., to 30 $H_{0}$ values.

The original ECG signal is plotted in Figure 5a. We report in Figure 5b the received signal ${ECG}_{received}$ after optical wireless transmission with an emitted power $P_{t}$ of 0.5 mW and a bit rate $R_{b}$ of 2.4 kbits/s. The recovered ECG signal ${ECG}_{analog}$ is plotted in Figure 5c.

It should be noted that the ECG signal is either restored or deteriorated, according to the evolution of the channel over time, i.e., according to the values of DC gain $H_{0}$. For example, the signal is strongly impacted at the beginning of the transmission and again between 4 s and 8 s, corresponding to the strong attenuations that occur over a long time interval. However, a substantial attenuation may not endure for very long because the channel can quickly change, depending on the trajectory. In this situation, it may be possible to recover the ECG signal.

These observations are based on an analysis of the morphology of the ECG signal, which is not reliable enough for clinical application. In order to deepen this analysis, in the following section, we evaluate the performance through SQI analysis to determine the transmission requirements, i.e., the transmission power, to maintain sufficient ECG signal quality during motion.

## 5. SQIs Analysis

### 5.1. ECG Signal and SQIs

An electrocardiogram is a non-invasive test used to measure the electrical activity of the heart. Variations in intra- and extracellular ion concentrations (which cause the myocardium to contract and pump blood through the body’s organs during each cardiac cycle) generate cardiac electrical signals [48].

Conventionally, 12 normalized leads exist for an ECG signal recorded with precise electrode position over the chest. Lead II is the most commonly used; it records the reference morphology of a normal ECG cycle, including five main waves: {P Q R S T}. This lead can be easily measured with only two skin electrodes, positioned near the right shoulder and on the left of the lower abdomen.

To interpret an ECG, the physician needs to focus not only on the frequency (heart rate) and regularity of the electrical signal but also on the shape and size of each individual wave, as well as the timing and interaction between waves. This is why the assessment of the quality of the ECG signals has become imperative with the development of portable ECG medical devices.

This aspect has been studied by different groups using SQI methods based on spectral, temporal, and statistical approaches [34,35,36,37,38,39,40,41,42,43,44]. In order to highlight the link between the conditions of an OWC communication and SQI evolution, we focus in this article on some SQIs in the frequency and time domains which are potentially affected by the transmission channel conditions.

For the spectral ones, we evaluated two basic features: the QRS complex quality and the high-frequency noise effects.

The distinct presence of the QRS complex in the signal is one of the main characteristics of a high-quality ECG. The QRS wave is generally concentrated in the ECG frequency band between 5 and 15 Hz, with a peak energy centered around 10 Hz. Thus, to provide a useful measure, we computed the QRS power spectrum distribution (*pSQI*), defined as the ratio between the QRS wave energy and the overall ECG signal [34]:

(7) $$pSQI=\frac{\int_{5\;Hz}^{15\;Hz}P\left(f\right)df}{\int_{5\;Hz}^{40\;Hz}P\left(f\right)df}$$

where *P*(*f*) is the power spectrum distribution of the ECG signal.

To assess the effects of high-frequency (HF) noise, which may occur due to strong variations in the transmission channel as the user moves around, we calculated the ratio of the power distribution between the useful ECG spectrum, which conventionally extends between 0 and 40 Hz, and the overall classical one, i.e., up to 100 Hz. This ratio was extracted based on the *HpSQI* defined in [42], and it is referred to as *xSQI* in the paper. *xSQI* is expressed as:

(8) $$xSQI=\frac{\int_{0\;\mathrm{Hz}}^{40\;\mathrm{Hz}}P\left(f\right)df}{\int_{0\;\mathrm{Hz}}^{100\;\mathrm{Hz}}P\left(f\right)df}$$

From (8), it is clear that when the *xSQI* is close to 1, the ECG signal is not impacted by high-frequency noise. On the other hand, low *xSQI* values mean that the power in the $\left[40–100\right]\;Hz$ band increases abnormally compared to the power distribution in the [0–40 Hz] band; this may be caused by HF perturbations contaminating the ECG signal.

To analyze the statistical features of the ECG signal, i.e., the Gaussianity and symmetry, we used the Kurtosis and Skewness functions, defined as [34,37]:

(9) $$kSQI=\frac{1}{N}\left|\sum_{i=1}^{N}{\left(\frac{x-\mu }{\sigma }\right)}^{4}\right|$$

(10) $$skSQI=\frac{1}{N}\left|\sum_{i=1}^{N}{\left(\frac{x-\mu }{\sigma }\right)}^{3}\right|$$

where x is the ECG signal, *N* represents the number of sample points, μ is the signal mean, and σ is its standard deviation (SD).

Skewness quantifies the degree of the distribution asymmetry of an ECG signal. This provides information about the presence of abnormalities or outliers in the ECG signal. However, it was demonstrated that the skewness of the ECG signal is sensitive to different sources of noise [40]. Kurtosis is also a statistical metric describing the shape of the distribution of an ECG signal. It provides information about peakedness and flatness in the ECG, relative to the normal distribution.

Both metrics are strongly dependent on the signal measurement conditions, the ECG leads, the types of noise, and the analysis context. Thus, it is difficult to define a general benchmark for these indexes. Empirically, this requires repeated observations and adjustments. Considering our approach in this work, where we adopted a pre- and post-transmission evaluation, we studies the evolution of the skSQI and *kSQI* with respect to the OWC communication conditions.

### 5.2. Methodology

**Figure 6:**
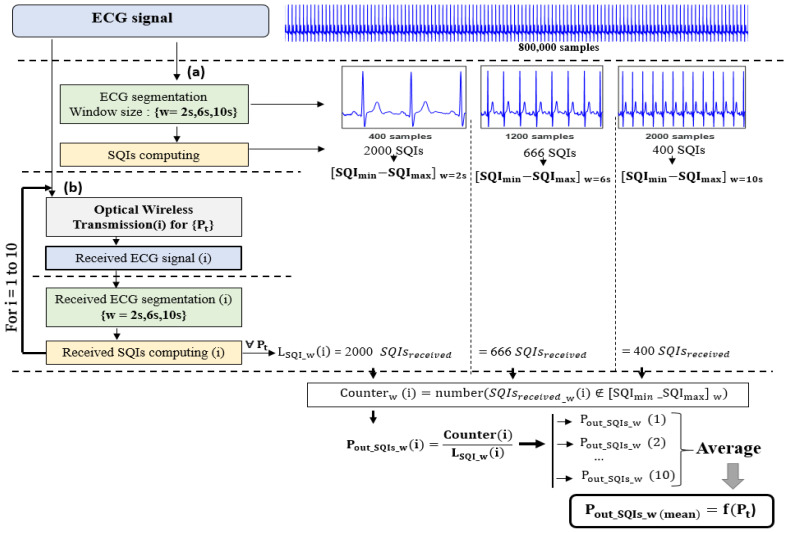
Flowchart of the proposed ECG assessment method: (a) Evaluation before transmission, (b) Evaluation after transmission.

Because of the lack of information in the literature about the reference identification criteria of SQIs, and because our objective was to study the impact of the performance of the transmission channel on the ECG signal, regardless of the acquisition conditions, we adopted a methodology consisting of evaluating the quality of the signal before and after transmission. The methodology flowchart is shown in Figure 6. It is composed of two main steps:

- Evaluation before transmission (refer to Figure 6(a))

Here, we evaluated the quality of the raw ECG signal before transmitting it by calculating the SQIs. With this aim, we used a windowing method which consists of dividing the signal of duration $T_{ecg}$ into non-overlapping segments of size w (refer to Figure 6(a)). This ensures accuracy in the quality assessment. Therefore, considering the elderly person’s walking trajectory described in Section 3.2, we used a corresponding ECG signal of duration $T_{ecg}=1\;h$. Then, the SQIs were calculated at each segment, so we obtained a set of $\left[\frac{T_{ecg}}{w}\right]$ SQI values varying into a range defined as $\left[{SQI}_{min}-{SQI}_{max}\right]$. This interval could then be used to define the criteria for identifying the SQIs as follows:

(11) $$SQI\;is\;excellent\;if\;SQI\in \left[{SQI}_{min}-{SQI}_{max}\right]$$

However, window size w over which we calculated the SQIs could have an impact on the results, depending on the requirements of the analysis, the specificities of the ECG signal, and the type of SQI. The larger the window size, the better the signal resolution for a good analysis and more stable estimation; however, a large window size can also introduce more noise and artifacts that impact the accuracy of the results. A smaller window size may provide a saving in computation time, but it does not guarantee a sufficiently accurate representation of the ECG signal and may even lead to false SQI values due to the increased amount of noise compared to the ECG data amount. In any case, a trade-off between window size and accuracy must be considered given the method used and the expected results. In order to account for these effects, we studied the evolution of SQIs over window sizes of w=2, 6, 10 s, as used in the literature [43] (refer to Figure 6).

**Figure 7:**
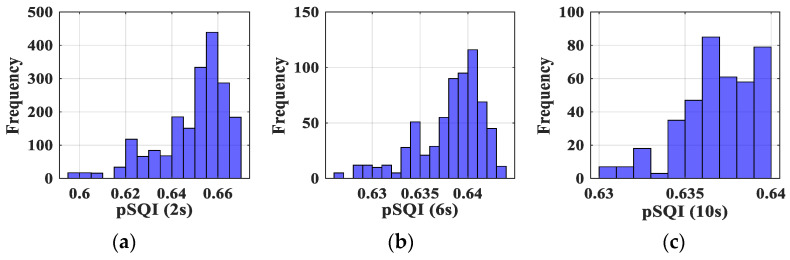
Distribution of obtained pSQI: (**a**) for a 2 s window, (**b**) for a 6 s window and (**c**) for a 10 s window.

As an example, Figure 7 shows the distributions obtained for the pSQI before transmission and according to the window size. We observe that, as expected, the range $\left[{pSQI}_{min}-{pSQI}_{max}\right]$ is lowered as window size w increases.

We thus determined the intervals $\left[{SQI}_{min}-{SQI}_{max}\right]$ for all the studied SQIs. The values are reported in Table 2. The same observation as for pSQI can be made regarding the impact of w.

- Evaluation after transmission (refer to Figure 6(b))

In the second step, we used the transmission chain detailed in Section 4.1 to recover the received ECG signal after it had been transmitted over the studied optical channel and evaluated its quality. For this purpose, we applied the same windowing method used before transmission and calculated the received SQIs at each window for which we obtained a set of received SQIs (refer to Figure 6(b)).

Then, in order to discuss the robustness of SQIs, we introduced the probability that the SQI does not respect the criteria, namely, the outage probability $P_{out\_SQI}$. Considering the identification SQI intervals determined in the previous step (before transmission), this probability was defined as follows:

(12) $$P_{out\_SQI}=P({SQI}_{received}\notin \left[{SQI}_{min}-{SQI}_{max}\right])$$

The lower this probability, the more robust the value of the SQI in relation to the transmission conditions.

### 5.3. SQI Robustness versus Emitted Power $P_{t}$

By varying the emitted power values, we studied the robustness of SQIs as a function of the optical transmission conditions. Based on the second step of our methodology (Figure 6(b)), for each $P_{t}$ value, the received ECG signal was segmented using the previously defined windowing method, i.e., by considering window sizes of w=2, 6, 10 s. Likewise, for each w value, the received spectral and statistical SQIs were computed. Then, from these received SQIs values, we used (12) and the results of Table 2 to determine for each $P_{t}$ value the corresponding outage probabilities. We repeated the process 10 times, as mentioned in Figure 6(b), and averaged the results to smooth the variations related to the randomness of the noise resulting from the optical transmission process.

**Figure 8:**
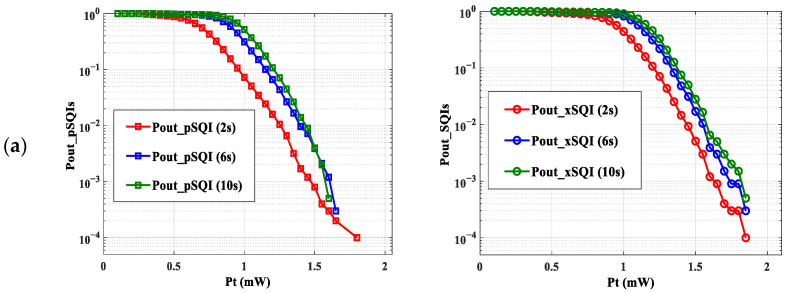

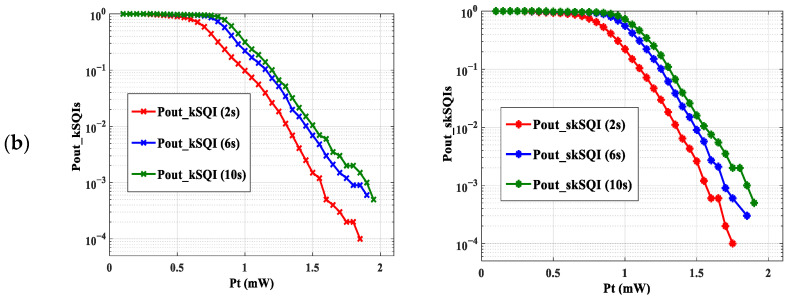
$P_{out\_SQIs}$ as a function of $P_{t}$: (**a**) Spectral SQIs, (**b**) Statistical SQIs.

For each window size, we reported in Figure 8 the evolution of spectral and statistical SQI outage probabilities as a function of $P_{t}$.

First, we can notice from Figure 8 that the shape of the curves is the same, regardless of the size of window w. As expected, the curves of $P_{out\_SQIs}$ decrease when the transmission power increases.

On the other hand, we note that for a given power, the smaller the size of the window, the smaller the value for $P_{out\_SQIs}$. As an example, focusing on the xSQI (refer to Figure 8a) and a $P_{t}$ of 1.5 mW, we can observe a $P_{out\_SQIs}$ of 0.56%, 1.9%, and 3.1%, for window sizes of 2 s, 6 s, and 10 s respectively. This can be explained by the fact that the SQI identification interval is larger when w decreases. Therefore, the probability that an SQI is outside this interval is lower.

From these observations, we can confirm that the window size on which we computed the SQIs can have an impact on results, and thus, it is necessary to choose the appropriate one to accurately assess the transmitted ECG signal.

**Figure 9:**
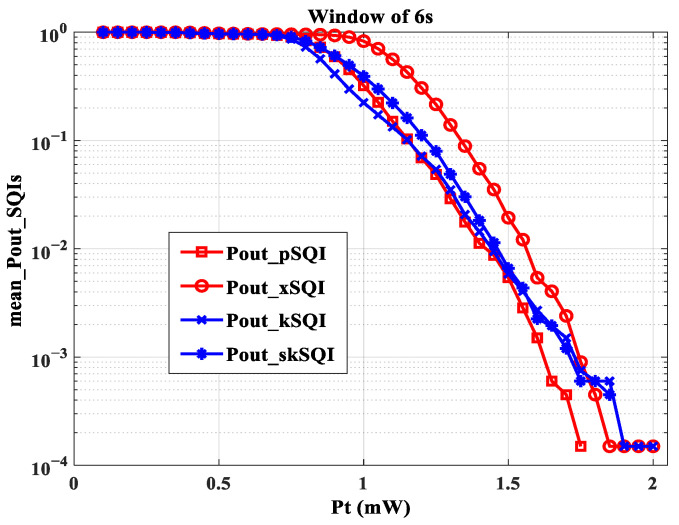
$P_{out\_SQIs}$ as a function of $P_{t}$ for a window size of 6 s.

Since our main objective was to investigate the impact of transmission performance of the proposed scenario, we focused on 6 s as an average window providing a good tradeoff between accuracy and computation time. Therefore, we reported in Figure 9 all $P_{out\_SQIs}$ as a function of emitted optical power $P_{t}$ for this window size.

We observed that the values of $P_{out\_SQIs}$ were lower and therefore better than for the other $P_{out\_SQIs}$. This would mean that the quality of the QRS wave, evaluated by the pSQI, is less impacted by the transmission conditions than the other characteristics of the ECG signal. This shows, for example, that it is possible to accurately measure heart rate, as defined by the RR interval, by using a wearable ECG connected with OWC. In addition, Figure 9 shows that the curves of $P_{out\_kSQI}$ and $P_{out\_xSQI}$ decline until they reach zero at a power value greater than those at which the other $P_{out\_SQIs}$ cancel out. This means that the two SQIs are more restrictive in terms of emitted power. Additionally, this finding was confirmed, regardless of the window size. This leads to a limit value $P_{t}=2\;mW$. Since it is essential to consider all SQI information, this means that with optical channel variations due to the proposed mobility scenario, at least a $P_{t}$ of 2 mW is needed to properly reconstitute the ECG signal. However, previously obtained results [31] showed that a power of 1.4 mW is required to obtain an acceptable ECG signal for a monitoring duration of approximately 6 min. The increase in power requirement in this work can be explained by considering more ECG SQI information during a longer monitoring duration (about 1 h) and by the improved evaluation method, which provides higher accuracy for analyses.

In order to determine the communication performance that corresponds to this $P_{t}$ condition, we determined the SNR and the BER using expressions (5) and (6). Considering the lowest gain obtained $H_{0}=-62.98\;dB$ (see Figure 3) and a $R_{b}$ of 2.4 kbps, an excellent ECG signal quality could be obtained for an SNR of 11 dB and, therefore, a BER of ${1.44\times 10}^{-4}$ with OOK modulation.

## 6. Conclusions

In this study, we considered the optical wireless transmission of the ECG of an elderly person walking in a room. The optical transmitter was located on the person’s wrist. Four optical receivers were arranged at the corners of a central panel in the ceiling of the room. In this context, the transmission channel was variable over time due to the random movements of the elderly during walking. From the distribution of the static gains of the optical link, we first observed the impact of the channel behavior on the temporal evolution of the received ECG signal, showing that it is necessary to take into account the performance of the transmission to assess the quality of the ECG. Thus, we have proposed a methodology to determine the impact of the OWC channel on several quality indexes (SQIs) which are typically used to evaluate ECG reliability. By comparing pre- and post-transmission SQIs using a windowing method, we have defined the criteria for identifying excellent SQIs. Then, the robustness of SQIs has been discussed by using the probability that the SQI does not respect the criteria as a function of the optical emitted power for OOK modulation. We have emphasized that all the studied SQIs reached their criteria of excellence at an emitted power of 2 mW. In terms of transmission reliability, this corresponds to a SNR of around 11 dB, corresponding to a BER of ${1.44\times 10}^{-4}$. Under these conditions, an excellent ECG signal could be obtained.

Regarding the mobility challenge, the obtained results are promising and less restrictive than the standard ECG signal specifications.

Furthermore, considering more challenging scenarios, such as a realistic environment with obstacles in the room, will likely lead to other conclusions and will be a challenge for future analysis. Therefore, the proposed evaluation approach could be improved by calibrating the used SQIs and incorporating more ECG features and appropriate classification methods to guarantee an accurate assessment of the ECG quality.

Moreover, to improve the system performance in terms of transmission reliability, power-efficiency, and implementing complexity, it is necessary to investigate other OWC modulation schemes. As example pulse position modulation (PPM) is more power efficient than OOK, but at the cost of increased receiver complexity. Another perspective for mitigating huge channel attenuations and probable blockages is to design and analyze a hybrid OWC/RF system and carry out performance comparisons between both technologies.

Finally, the proposed generic framework we provide in this work is suitable for any monitoring scenario where the transmission channel conditions can impact the ECG signal. It is applicable regardless of the setting and the evaluation indexes used.

## Figures and Tables

**Table 1:** Summary of developed ECG monitoring systems using OWC technologies [17,18,19,20,21,22,23,24,25,26,27,28,29,30].

References	Optical Band	Link Configuration	Transmission Metric	ECG Evaluation Method
[17] 2013	Visible/VLC	LOS in static scenario	Experimental PER = 1.3% over 50 cm	ECG shape and Heart Rate checking
[18] 2014	Visible/VLC	LOS in static scenario	Experimental PER = 2% over 1 m	ECG shape and Heart Rate checking
[19] 2015	Visible/VLC	LOS in static scenario	_	Experimental Mean Square Error = ${3\times 10}^{-3}$ over 2.4 m/Experimental Mean Square Error = ${3\times 10}^{-3}$ over 2.4 m and ECG shape checking
[20] 2016	Visible/VLC	LOS in static scenario	Experimental PER = 0.5% over 1 m	ECG shape and Heart Rate checking
[21] 2017	Visible/VLC	LOS in static scenario	Analytical $BER>10^{-6}$ for analytical SNR of 14 dB	_
[22] 2017	Visible/VLC	LOS in static scenario	Experimental PER = 0.0005% over 11 m (d = 11 m, using Fresnel lens)	ECG shape and Heart Rate checking
[23] 2017	Visible/VLC	LOS in static scenario	Experimental PER > 0.005% over 6m (using plano-convex lens)	ECG shape and Heart Rate checking
[24] 2019	Visible/OCC	LOS + NLOS in mobility scenario	_	Comparison between original and transmitted Heart rate
[25] 2019	Visible/VLC	LOS + NLOS in mobility scenario	Experimental $BER=1.2\times 10^{-6}$ over 1.25 cm	ECG shape and Heart Rate checking
[26] 2019	IR	LOS in static scenario	Analytical $BER=10^{-6}$ over 0.75 m	ECG shape checking
[27] 2019	Visible/OCC	LOS in static scenario	OCC outage probability of $10^{-2}$ over 2 m	_
[28] 2020	IR	LOS + NLOS in mobility scenario	Experimental PER = 8.6% over 5.2 m	ECG shape and Heart Rate checking
[29] 2022	Visible/VLC	LOS in static scenario	Measured receiver sensitivity of −30 dBm for good system performance (92% of accuracy)	Comparison between the shapes and the HRV values of the emitted and the received ECG
[30] 2022	IR	LOS+NLOS in mobility scenario	Analytical SNR of 10 dB and required power of 13 mW for good ECG quality (all SQIs respecting thresholds)	Comparison between original and transmitted ECG shapes/Extraction of statistical and spectral SQIs/SQIs as a function of SNR

**Table 2:** SQI identification criteria for each window size.

Window Size *w*	*pSQI*	*xSQI*	*kSQI*	*skSQI*
2 s	Min = 0.597 Max = 0.667	Min = 0.986 Max = 0.998	Min = 13.975 Max = 21.686	Min = 2.611 Max = 3.49
6 s	Min = 0.626 Max = 0.643	Min = 0.994 Max = 0.998	Min = 15.837 Max = 18.231	Min = 2.871 Max = 3.147
10 s	Min = 0.63 Max = 0.639	Min = 0.996 Max = 0.998	Min = 16.247 Max = 17.683	Min = 2.924 Max = 3.08

## Data Availability

Not applicable.
